# Assessment of the use of Open Educational Resources at five European Library and Information Science higher education institutions during and post-COVID-19 pandemic

**DOI:** 10.12688/openreseurope.17457.2

**Published:** 2024-09-09

**Authors:** Gema Santos-Hermosa, Cristóbal Urbano, Sílvia Argudo, Juan-José Boté-Vericad, Anja Đurđevic, Milijana Micunovic, Lea Wöbbekind, Tania Todorova

**Affiliations:** 1 Departament de Biblioteconomia, Documentació i Comunicació Audiovisual, Universitat de Barcelona, Barcelona, Catalonia, 08014, Spain; 2 University Computing Centre, University of Zagreb, Zagreb, City of Zagreb, 10000, Croatia; 3 Faculty of Humanities and Social Sciences, Josip Juraj Strossmayer University of Osijek, Osijek, Osijek-Baranja County, 31000, Croatia; 4 Institute for Information Science and Natural Language Processing, University of Hildesheim, Hildesheim, Lower Saxony, 31141, Germany; 5 University of Library and Information Technologies, Sofia, 1784, Bulgaria

**Keywords:** Open Educational Resources (OER), Library and Information Science (LIS), Higher Education, COVID-19, Emergency Remote Teaching (ERT), Digital education.

## Abstract

This article presents an analysis of the impact of Open Educational Resources (OER) during the COVID-19 pandemic, as well as their potential use in the post-pandemic, in Library and Information Science (LIS) higher education institutions. The research explored how OER were used and created, what were the main barriers and drivers in practice and some main lessons learned that can help to improve the quality and increase the use of OER beyond times of crisis. The research was based on fieldwork carried out in the LIS departments of the universities of Barcelona (Spain), Hildesheim (Germany), Osijek and Zagreb (Croatia) and the University of Library Studies and Information Technologies in Sofia (Bulgaria). The methodology approach was qualitative and was based on interviews with faculty and focus groups with students. Results show that faculty members were still hesitant to adopt OER since they generally did not consider them. Moreover, those who did use them did so on their own initiative and as additional resources. We discuss the different speeds of OER implementation that have been observed depending on the faculty’s prior level of knowledge, and on whether their institutions and countries are prepared to support the use of OER. The promotion of post-pandemic OER involves greater capacity building, as well as collaboration and institutional support. Students’ attitudes about the usefulness of OER focus on their availability. The large number of teachers and students who participated in the study, as well as the international scope of the study, constitute a strength in the treatment of a topic such as the use of OER where the user perspectives and LIS context have been little addressed in the literature.

## Introduction

The global COVID-19 pandemic impacted education in a disruptive way, forcing academics and students worldwide to adapt their courses to the online educational environment in a short time. This situation led to the adoption of more online resources than ever before; ranging from paid resources to commercial content that was temporarily available for free during the pandemic and Open Educational Resources (OER) ( Schaffhauser, 2020; Stracke *et al*., 2022).

While there is no single definition of OER, the current UNESCO definition -included in the UNESCO OER Recommendation- is widely accepted: “learning, teaching and research materials in any format and medium that reside in the public domain or are under copyright that have been released under an open licence, that permit no-cost access, re-use, re-purpose, adaptation and redistribution by others” ( UNESCO, 2019, 5). In such an urgent transition during the pandemic to remote education, OER might be very important given their characteristics of openness, low cost, and flexibility. Moreover, they have the potential to transform teaching and learning to make these more accessible and equitable ( Miao *et al*., 2016). However, different conditions must coincide in the space and time to make them genuinely convenient and useful in a crisis of such magnitude. To help determine and examine the real impact and usefulness of OER, this article aims to contribute with a European case study based on the experience of five higher education institutions (HEIs) in the field of Library and Information Science (LIS).

### OER global initiatives

The pandemic increased awareness of the internationalization of educational resources, which led to the development of open education solutions and platforms and more sharing and use of OER around the word.

Research into the impact of OER during the COVID-19 pandemic, and beyond, can be approached from different perspectives, (technological, sociological, emotional and economic). The one that we are firstly interested in within the scope of this study is how OER have been introduced and used to support emergency remote teaching (ERT) in higher education (HE)

In this sense, there are several reports from international organizations ( European Commission, 2020; OECD, 2021; UNESCO, 2022) that show worldwide examples of positive initiatives and changes in education as a reaction to the pandemic, both at the institutional and national levels. There is also some collaborative research, based on analysing case studies in different countries ( Biernat *et al*., 2022; Bozkurt *et al*., 2020; Ossiannilsson *et al*., 2020; Stracke *et al*., 2022), that provides a global outlook to assess the COVID-19 education crisis in relation to OER. 

Some studies highlight that countries with years of experience in distance learning systems (such as in India, South Korea, and Sweden), or with a long tradition to Open Education (as Canada) and HEIs with existing OER repositories or communities of practice based on the ideology of Open Education, were better prepared for the emergency switch to remote education that the pandemic entailed ( Biernat *et al*., 2020; McGreal, 2020; OECD, 2021; UNESCO, 2022); since they had several initiatives that supported the use and adoption of OER.

For instance, there were countries, such as China, Brazil and Indonesia, that had OER government-initiated and supported programmes ( Huang *et al*., 2020; Ossiannilsson *et al*., 2020; Alhaj *et al*., 2021). At an institutional level, there were also examples of online universities (such as Athabasca University, Open University of the Philippines and Indira Gandhi International Open University) and others with previous experience in online learning or open education (such as British Columbia) that were aware of OER and were using them during the pandemic ( Arcebuche, 2022).

Most of this relevant literature collects cases of positive reactions to COVID-19 lockdowns through the implementation of OER in education, but it does not delve into what happened in those countries or institutions that were less prepared or had less experience.

### Main actors, contexts and scenarios of OER

The focus on OER has been approached from several different geographies and disciplinary angles and has considered various stakeholders. Our study aims to understand the use of OER in a specific spatial context and disciplinary field, that of LIS at European level, and with the focus on faculty and students.

A systematic review by Otto *et al*. (2021) reported about the little-investigated empirical effects of the use of OER on established pedagogical approaches. Nevertheless, several studies have been identified on the faculty’s perspective regarding general awareness of OER ( Biernat *et al*., 2021; Christoforidou & Georgiadou, 2021; Watermeyer *et al*., 2021) and their attitudes to OER use ( Karataş *et al*., 2022; Otto, 2022; Sunar *et al*., 2022). Regarding the students’ view, apart from a few projects that focused on their perceptions of the usefulness of OER ( Arcebuche, 2022; Cheung *et al*., 2022), most have centred primarily on the students’ attitudes towards technological change in education ( Aguilera-Hermida, 2020; Händel *et al*., 2022).

A systematic review by Otto *et al*. (2021) reported about the little-investigated empirical effects of the use of OER on established pedagogical approaches. Nevertheless, several studies have been identified on the faculty’s perspective regarding general awareness of OER ( Biernat *et al*., 2021; Christoforidou & Georgiadou, 2021; Watermeyer *et al*., 2021) and their attitudes to OER use ( Karataş *et al*., 2022; Otto, 2022; Sunar *et al*., 2022). Regarding the students’ view, apart from a few projects that focused on their perceptions of the usefulness of OER ( Arcebuche, 2022; Cheung *et al*., 2022), most have centred primarily on the students’ attitudes towards technological change in education ( Aguilera-Hermida, 2020; Händel *et al*., 2022).

From a disciplinary perspective, Cisel (2024) notes the need to consider the academic discipline of faculty and students when exploring their experiences with OER. In this regard, LIS knowledge area is singularly interesting since there are few studies that directly address OER in this discipline and fewer that refer to their impact on faculty and students during or after the COVID-19 crisis. Among those that do exist, some predate COVID-19 ( Katz, 2020) or focus on parallel subtopics such as LIS training programs ( Bell, 2021; Santos-Hermosa & Atenas, 2022) and the role of the librarians in OER ( Kimball *et al*., 2022). Finally, there is another group of LIS studies that address OER from a broader perspective; mainly from digital education ( Wöbbekind *et al*., 2023) or awareness ( Sibiya & Evans, 2024)

Otto *et al*. (2021) provided evidence that the research on OER had been, mainly, located in North America. Priora & Carloni (2023) also added that the European scenario on OERs looked rather different from US and Canadian realities. In this regard, the European Commission (2020) highlights that European universities were moving first steps towards the promotion of OERs and that massive open online courses (MOOCs), offered by some European HEIs, aroused great interest during the pandemic. Other more recent European projects, such as the European Network for Catalysing Open Resources in Education (ENCORE) reported that, despite the ambition of the EU to achieve an open and inclusive educational environment (Europea Education Area) by 2025, the uptake of OER in Europe still appeared to be a scattered and disharmonized phenomenon ( European Network for Catalysing Open Resources in Education, 2021). 

This scenario shows that the pandemic has exacerbated inequalities both within and between countries, also it has revealed positive features and initiatives as well as some weaknesses and vulnerabilities. For instance, copyright rules in the EU are still significantly fragmented, copyright legislation presents one additional disadvantage ( Priora & Carloni, 2023).

To fill in some of the aspects pointed out by the previous literature, as well as potential research gaps in research like evidence of OER use by less prepared institutions – such as, predictably, DECRIS partners –, within the European disparity of approaches to OER and the scarcity of research in the LIS area, our research aims to contribute to the understanding of existing practices regarding the use of OER as well as its advantages and disadvantages, during the pandemic and beyond. More specifically, and as already mentioned, from the perspective of academics and students from five European higher education institutions in the LIS discipline.

### Purpose and research questions

We consider that the value of this paper is that it not only identifies the main barriers, as well as the benefits of adopting OER, but also highlights some real-life practical experiences of these resources during the ERT in the COVID-19 pandemic. Moreover, it also provides useful guidelines on how to effectively integrate OER beyond the pandemic period, based on what has been learned from these experiences. To explore and discuss all these issues, we established three research questions:

Q1: In what ways have OER been used and created for ERT during the COVID-19 pandemic?
Q2: What are the barriers and advantages of OER reported during the COVID-19 pandemic?
Q3: What are the main lessons learned during the pandemic that can help foster OER post-pandemic?

## Methodology

To describe the design of the fieldwork and data analysis, we have checked it against the Consolidated Criteria for Reporting Qualitative Research (COREQ) ( Tong *et al.*, 2007). In this section we present different aspects related to research team and reflexivity, study design and data analysis and reporting.

A qualitative approach has been adopted through semi-structured interviews with faculty and focus groups with students, all from LIS departments at four European countries of the five HEIs that are partners of the DECriS Erasmus+ Project. Content analysis was chosen as the theoretical framework to systematically organize data into a structured format through inductive open coding. Both, interviews and focus group methodologies, are well-established methodologies in educational science. While the first allows individuals to explain how they understand and interpret the world around a topic ( Brinkmann & Kvale, 2018), the second provides interactions within a group and generates both different and common feedback and ideas ( Smithson, 2000).

During the first four-month period of 2022, a total of 39 interviews with faculty and 10 focus groups with a total of 47 students were carried out, both face-to-face and online, depending on the COVID-19 situation or the participants’ preference. No one else besides the participants and researchers was present during the interviews and focus groups.

The interviewers and moderators of the focus groups were part of the working teams from each of the institutions participating in the DECriS project. They were all faculty from these institutions and received specific training in order to take consistent testimonies from the 5 institutions where the fieldwork was carried out. They were all familiar with the context and the profiles of the interviewees, given that they belonged to the same institution. The interviewees were well informed about the DECriS project and the research objectives. The fact that the interviewers and interviewees belonged to the same institution was an advantage, as there was a common basis of knowledge of the context of each of the institutions. On the other hand, this familiarity between interviewers and interviewees may have been a limitation in terms of the way in which interviewees expressed their experiences and opinions.

The breakdown of participants by partner can be seen in Table 1. Throughout the paper, all the quotes from the interviews are labelled with the partner code followed by the interview number (e.g., [OJ_1] for Osijek teacher #1); for focus groups we added the letters FG before the number (e.g., [OJ_FG_1] for Osijek focus group #1).

**Table 1.** Number of interviews and focus groups held by all partners.

Partner	Interviews	Focus Group (Students)
Barcelona [BA]	14	2 (10)
Hildesheim [HI]	3	2 (9)
Osijek [OJ]	9	1 (6)
Sofia [SO]	8	4 (16)
Zagreb [ZA]	5	1 (6)
Total	39	10 (47)

As we used two qualitative techniques that involved a relatively small number of people compared to the total teacher and student populations, it was necessary to determine the criteria and the sampling system to decide who would be interviewed and who would be part of the focus group meetings. Therefore, the purposive sampling technique (i.e., non-probabilistic sampling) was applied under the knowledge that each partner had of the context of their participants. To simplify the conduct of the interviews and focus groups, once the criteria and the sampling routine had been established, each partner institution made its choice of people considering that it was necessary to ensure that the samples had the appropriate representation regarding the different students’ levels and teachers’ subject domains. The following criteria were applied:

- Bachelor’s and master’s degree faculty and students of various courses who were teaching or enrolled during the pandemic period of lockdowns (2020–2021).
- Parity between genders and age groups was respected both for the participants of the interviews and the focus groups.

In compliance with the COREQ guidelines for interviews and focus groups, our research prioritized ethical considerations. Written informed consent was diligently obtained, ensuring participants understand the study's purpose. Stricter measures, including interviewees quotations anonymization at the results outputs and secure data storage, were implemented to guarantee confidentiality. The design of the study, as well as the process of data collection and its safekeeping, was approved, on 2021-11-05 by the Bioethics Commission of the University of Barcelona (CBUB) [Institutional Review Board (IRB00003099) http://www.ub.edu/comissiobioetica/en], as the Barcelona team led the IO-2 work packge of the DECriS Project.

The interviews and focus groups were conducted and audio-recorded, following common guidelines ( Urbano *et al*., 2024), by each education institution in the native languages of their respective countries (Bulgarian, Catalan, Croatian, German, and Spanish). The audio recordings were transferred to the Barcelona team to carry out an automatic transcription, which was subsequently edited, listening to the original audio and translated into English by each of the participating teams. During the editing and translation process, the most important phrases and key concepts were highlighted by each partner team, to guide the coding work by the Barcelona team who consolidated the report on the interpretation of the results. Qualitative data analysis software, Atlas.ti, facilitated the management and analysis of interview and focus groups transcripts. With all the text files in English, the Barcelona team worked on the inductive coding ( Mayring, 2010) of all the transcriptions and data analysis to produce the preparation of the reports of each centre and the final joint report. The analysis was double-checked by a researcher of each partner to avoid bias or misunderstandings. The questionnaire script used in the interviews and focus groups was structured, in both cases, into six main thematic sections with a total of 38 questions which is also included in the Mayring approach ( Boté-Vericad *et al*., 2022; Braun & Clarke, 2006). While this guide covered a wide range of topics, aligned with the overall objectives of the DECriS project, this paper focuses specifically on the topic referring to the real situation and perception of OER. Table 2 presents the relevant general coding scheme (four of the six main themes relevant for our study: Adaptations, Problems, Advantages and Improvements) and, in parentheses, the specific themes for this specific OER section; connected to our research questions. 

**Table 2.** Traced occurrences for “OER” codes.

Code	Subcode	Category
Adaptations-AD (OER Use and creation	AD01	Approaches to emergency remote teaching
	AD04	Supplementary materials provided by teachers ( *)
	AD05	Looking for new educational resources and use of OER
	AD06	Creation of OER
	AD08	Production of videos or voice clips other than recorded live lectures
Problems-PR (OER Barriers)	PR06	Not a good time to create new material from scratch purposely designed
	PR08	Access to library resources and copyright problems
Advantages-AV (OER Advantages)	AV04	Promotion of the creation and reuse of educational resources
Improvements-IM (Lessons learned)	IM03	Promotion and support for improving OER use and creation
	IM05	Teachers’ training and mindset change
	IM08	Improvement of the students’ workload estimation when planning courses ( *)
	IM15	Strengthening the role and resources of libraries and support services for teachers

Note: Those categories with (*) refer just to students

## Results

### OER use and creation

In terms of how the OER were used, faculty reported that they adapted previous materials to the online environment and that some of them also used OER as additional or supplementary material. They specified that they used open resources from others (such as videos, TED talks, MOOCs, etc) and resources in the public domain, as can be seen in the interview extracts below.

“I have used some of them [OER], but they were not my own resources. I've already forgotten what they’re called – a kind of online university where you have MOOCs”. [HI_8]
  “I use mostly video materials because I think the students are already reading enough written texts. They were mostly video lectures that were in the public domain or available openly on different channels like YouTube” [OJ_6]
  “[Looking for OER] Well, maybe some videos, or some TED talks that I later asked the students to comment on, or I would ask them questions related to the videos.” [ZA_5]

It stands out that, while in some cases the faculty verified the terms of use of the material and whether it was available under the open licence, in others they did not:

“I approach these resources very carefully […] I avoid using resources directly without checking the status of the material.” [SO_7]
  “I developed presentations, especially for online learning, in which I put links to access various interactive applications as additional materials with open access. I didn’t check whether the resources are licensed or open for use, but after they were uploaded for free access on the Internet, I assumed that training resources were open.” [SO_8]

In addition, it has been observed that some faculty members also introduced GLAM (galleries, libraries, archives and museums) resources into their teaching, although it is not clear whether these were OER or non-OER (i.e. with subscription or not under open licences or as public domain with clearly labelled).

“I especially warn my students to be very careful when using Internet resources and direct them to verified sources. I advise them to use the opportunities offered by libraries and museums.” [ *SO_7*]
  “Because knowledge exists to spread, I also introduced materials and resources provided by various Bulgarian institutions - libraries, archives, museums, etc., as well as promoted access to the digital library of the University, which we created in connection with the pandemic and its consequences.” [ *SO_8*]

On the other hand, some students reported that they turned to YouTube on their own for additional help, from tutorials for instance, to fill the gaps in the faculty’ guidance:

“... if we passed the subject, it was because of the YouTube tutorials not because of the teacher. So, you couldn't record his class to follow it as a tutorial, you couldn't do the exercises while he was doing them because you'd miss what he was doing on the screen.” [BA_FG_1]

Concerning OER creation, faculty said that they produced short videos to clarify very specific issues or to give instructions on installing and using software, using information resources, etc. The online environment also led to more project-based learning activities and gamification resources being developed.

“It's a lot of work to record them [videos] and it took a long time. […] But of course, you have to think about the fact that you can reuse them” [ *HI_4*]
  “We actually developed some virtual projects. For example, a virtual museum of students and their works in publishing, where they were very engaged.” *[OJ_2]*

Another phenomenon observed in the interviews is that although the faculty endorsed the OER philosophy, their productions were not always conceived as such - they did not label them as OER or consider they had OER characteristics.

“I'm not sure to what extent [I created OER], because, of course, personal experiences are one thing, yes, and the possibility of exchanging [teaching experiences], creating Creative Commons materials, for example, and having them available in a community of users, is another. [BA_8]

In addition, it seems there was not a formal way to integrate OER due to the costs of a more elaborate preparation for the public presentation of materials and the creator's belief that the resource is not good enough or not fully finished; as well as challenges related to licensing, depository, indexing, etc.

“My only concern [creating OER] is that it's not the best quality. And then I think it would be stupid if someone uses it and then is somehow disappointed […]. So if it didn't cost anything, I would probably say, well, don't cry, if something doesn't work, it's free. But then I would have it. I would like to have it in such an optimal condition, which of course would require a lot of work and refinement.” [HI_4]

Opposing positions can be seen regarding the suitability of the pandemic times for creating OER. Some faculty members stated that it was not a good time to create new material from scratch designed for a specific purpose, since the course was ongoing and was not planned to be carried out in a crisis situation, such as the ERT.

“I didn’t create new materials so as not to change the students’ work dynamics too much. In fact, we tried to work much harder to support these materials than to create new ones.” [BA_10]

Others, however, considered that some experiences and resources used during the health crisis stimulated the creation of materials, some of which may continue to be useful in the return to a more normal situation beyond COVID-19.

“I kept all the materials somewhere in my archive, so in case we had to teach online again some time, then I could access the materials again. […].” [OJ_4]

### Barriers to OER

Some of the main problems that faculty reported were about OER concern findability issues, the lack of knowledge or awareness about OER and the lack of OER other than foreign language resources.

In terms of the problem of finding OER, we observed that some faculty searched for OER with the aim of facilitating more dynamic and autonomous learning, but they were not successful. They considered that this was because there were not enough repositories of OER, there was no specific content ready for teaching and currently there is not a culture of sharing:

“This is about looking for other digital materials... In fact, I had already looked for them before [the pandemic], but I hadn't managed to find them. [...] We should have more repositories of materials that are useful to us... [the problem is that] we don't share them, or we don't know how to find them.” [BA_12]
“I had problems finding some of the materials and resources that I could use […] I think we should have more repositories of OER and different digital materials that are available to use so there are more in the public domain or licenced through Open Licencing by CC. And I think we should be more open to sharing resources. We talk about open science and open education, but in practice we don't see it so much.” [OJ_6]

Searching for videos and online material available in open access, not previously used in face-to-face teaching, has been identified as an important trend among some faculty to adjust their teaching planning. While some did not find enough online bibliography and digital material openly available, others found videos on YouTube or TED talks from key authors whose books and articles they had recommended in the subject bibliography.

“I looked for more bibliography online, [a search] that I might not have done in the case of normal face-to-face teaching.” [BA_1].
  “I am currently preparing a new subject. I listen to these video lessons by Russian colleagues, as well as North American ones, because I have their books. I have downloaded them for free, there is free access, but I saw that they also had these video lessons that were available on YouTube.” [SO_11]
  “I use open access platforms such as TED and YouTube, where the authors have voluntarily provided their materials for free use. I encourage students to use the opportunities of libraries and museums, which provide virtual tours, digital collections and online information services.” [SO_7]

In terms of not knowing about or being aware of OER, the express creation of OER was absent in most of the faculty’ discourses and reflections. Faculty did not differentiate between open digital materials and the educational resources that correspond strictly to the set of characteristics that define OER.

One of the weaknesses of OER identified through the interviews was that they are not part of faculty’ mental framework. Faculty do not know their distinctive characteristics or the repositories in which to locate them and how to evaluate them. In some cases, in which OER were specifically mentioned, faculty said that the ones they found did not fit what they really needed. It is observed that most of them had a fuzzy idea of what OER are and where to look for them.

“I’ve probably used them [OER]. Yes, when I used them, I was interested, but now I can't say anything specific.” [SO_2]
  “Probably the first thing that came to my mind was that I probably didn't even think that there could be anything relevant to my course, since the topic is quite specific, but I don’t know.” [ZA_1].
  “I suppose that faculty should investigate the OER available to make more use of the resources that already exist out there ... But, of course, you start searching from your personal experience of contents created for a face-to-face class. Finding equivalents that refer to that exact content is very difficult.” [BA_1]

The lack of knowledge that has been identified is also linked to a lack of time to look for new solutions or to adapt existing ones.

“It's hard for me to find things that are useful. You spend a lot of time looking and what I find isn't much...I think that the idea of OER is very good, but in practice it's hard for me to apply it.” [BA_12]

Finally, the lack of non-English OER or availability in multiple foreign languages are other barriers that faculty report.

“The problem is that they [OER] are in English. Yes, there are opportunities for automatic translation, but the quality is not good enough. A big problem with my subjects is that there are no similar materials for the Bulgarian archival institutions.” [SO_4]

### Benefits of OER

Some faculty members pointed out that some of the main advantages of OER are that they encourage and foster reusability of materials, that they can be adapted and enriched, and they are available and accessed quickly:

“Another interesting topic when educational materials are adapted for online teaching is that it encourages and fosters reusability of materials. So, if you can reuse it, then I can also be sick for a week and just upload it.” [HI_4]
  “The main advantages of the OER that I would point out are the fast access and that is easy to make changes. I can edit something, I can add, enrich, adapt the resource. They are available from anywhere at any time.” [SO_1]
  “I have always believed that what I develop for my courses should be accessible to a wider range of users; so, yes, this was always my attitude. And now the pandemic has intensified this feeling and I think it would be good to work towards creating OER in Bulgarian.” [SO_6]

Although there is no evidence whether students are referring to OER or general digital resources, they express a need for some kind of material that they can reuse asynchronously outside the classroom for self-paced learning, to adjust their learning paths, and for clarification, etc.

“Prof. xx pre-recorded her lectures, so we could listen to them whenever we wanted. The great thing about it is that you can go back to it and listen to the lecture as much as you want.” [OJ_FG_1]
  “Apart from googling and searching for materials when we need to find additional information or clarify something we didn't understand, I haven't used any special tools like this [OER], except for normal databases, articles and similar things.” [ZA_FG_1]

### Needs and requirements to foster OER: lessons learned

Throughout the interviews, it was observed that there have been few approaches to ERT that involved OER, and these have differed among DECriS partners. Most faculty stated that they modified their materials to make them more compatible with the online environment, but there was little emphasis on specifically including OER. However, there were some cases of institutions and faculty who were more familiar with e-learning (since they had previous experiences and a positive attitude) and were therefore more likely to use OER and implement them in their teaching practice.

The focus groups revealed that although students were happy with the availability of more digital resources, they wanted the teaching of the subjects to be planned better and for the pedagogical practices to be more innovative.

In this diverse scenario caused by the pandemic, some lessons were learned about what requirements were necessary to promote OER beyond the crisis. According to faculty, a change in mindset and more awareness, together with more training and new skills, were some of the necessary conditions. Although institutions offered training courses, they were more related to the features of the Moodle platform associated with active teaching and educational technology, rather than specifically on creating and using OER.

“Everyone needs new competencies. So, faculty' competencies are very low.” [OJ_2]
  “Perhaps it would not be a bad idea to offer some kind of education in general on producing new teaching materials. So that each teacher can choose what suits them.” [OJ_1]
  “I have the feeling that it is difficult to deviate from it or to try something new, and we're going through a period of upheaval […]. Something is changing, simply because many new, younger faculty are coming in who are also willing to try something new.” [HI_8]
  “We should work more on building awareness around OER. Because I think it's a general problem, so people aren't producing or using OER enough.” [OJ_6]

During the interviews, other needs also came up, such as the creation of a catalogue, a guide, or a list of quality teaching material for the different subjects, as well as tools to be able to use the technology better (videos, subtitles, etc.). With a few exceptions, faculty also indicated the lack of centralized support and resources from their institutions and how an improvement in this support and more collaboration could benefit the use of OER. For example, it would be useful to have directives, to share good practices and solutions to problems experienced while using tools and have more resources and digital platforms.

“[We need] more technical directives about tools, less dispersion…. less "do it yourself"[...] more centralized [support is needed], more training for faculty on resources to work online with a different model of courses.” [BA_14]
  “I think it [fostering OER creation] should come from the top, I mean, the university is still a very hierarchical system, and as long as there is no directive from the top that says we want to do this, it’s difficult.” [HI_8].

## Discussion

At the beginning of our study, we had assumed that, in crisis situations, OER would be a key resource for online teaching. But all indications are that this assumption as a starting point was not correct. That is, if OER were not even minimally present in the mental framework or current routines of teachers in normal situations before COVID-19 (as direct users, recyclers or creators), OER was unlikely to be considered in crisis situations. This is why differences in the use of OER were observed according to the degree to which individual institutions in our study, or subjects within an institution, were more digitally and computer oriented. For example, compared to the other institutions, the prevalence of OER use was higher in Hildesheim (Germany), in many curses involving student work with computer applications for which there are materials available at repositories like OpenHPI, that fall within the definition of OER.

But in general, for similar academic subjects, we have not actually identified many different perceptions of faculty and students towards OER depending on their context. In any case, for all the five institutions, the more digital and computer oriented was the academic subject or the teacher background, the more likely OER could have some presence during the ERT and beyond. From a general perspective, as demonstrated with our field work, the presence of real OER has been very low in the solutions adopted in response to the shift to various forms of online or hybrid teaching due to the pandemic. In short, OER are not a magic “pill” to lower the challenge of organizing the ERT alternative to normal teaching. OER require exploration, evaluation, design, adaptation and a final fitting to the academic subjects; tasks that were impossible to carry out due to the rush of the first lockdown and the uncertainty of the following semesters under COVID-19.

The faculty interviewed mostly reported that they did not use OER because they were not familiar with the term, OER were difficult to locate, or they did not consider it relevant (because of the language or because they were not ready for specific teaching or discipline needs). However, those who did use OER generally did it as an individual initiative (not supported by the institution) and as additional or supplementary material within their teaching. This situation does not seem isolated, since other European project ( European Network for Catalysing Open Resources in Education, 2021) also identified faculty who reported that their organizations' level of experience in OER adaptation was low or even non-existent. We therefore consider that the overall profile of faculty from DECRIS institutions would fit the "activist" category, which according to ENCORE would be characterised by being open to using and sharing OER altruistically, without relying on encouragement from their organisation or direction provided by institutional policy.

Our findings on the barriers to using OER show that they are aligned to other studies. More specifically, the lack of awareness about OER ( Seaman & Seaman, 2017) and the legal uncertainty ( Otto *et al*., 2021) have been found to be the major obstacles to using OER. In this respect, a related key aspect emerged from our results and present in other literature is the urgent need for professional development and training for faculty ( Santos-Hermosa & Atenas, 2022; Stracke *et al*., 2022), as well such as not focus on the use of e-learning tools but rather on how to create open content ( Nabukeera, 2020).

Moreover, the findability problem connected to the experiences of it being time-consuming to locate relevant OER in different repositories was also a common impediment reported by faculty ( Biernat *et al*., 2022; Otto *et al*., 2021), despite the growing number of OER in repositories ( Santos-Hermosa *et al*., 2021). As there is no centralized infrastructure for sharing OER, which has been reported as a widespread problem worldwide ( Stracke *et al*., 2022), one possible measure would be to have a meta-searcher ( Otto *et al*., 2021) to facilitate access from one single platform. In this sense, LIS professionals (both faculty-students binomial and academic libraries) might have a critical role in strengthening action plans and infrastructures to ensure the discoverability of OER.

LIS faculty also stated that the lack of OER for specific topics or perspectives, and the lack of culturally or linguistically tailored OER, were other issues that contribute to these resources not being used. This specificity can be seen as a paradox, since the potential of OER is precisely the flexibility to be adjusted locally and globally. OER have the advantage that faculty can adapt them to their own context to maximize student engagement and promote diverse learning experiences ( Van Allen & Katz, 2020). In any case, our findings are aligned to Koseoglu *et al*. (2020), who note a lack of OER development in non-English languages and the need for local OER to fit the learners’ contexts and represent proximity perspectives. Our results also underline the need to have subject-specific OER in the LIS discipline, in accordance with the study by Christoforidou and Georgiadou (2021) limited to the field of Arts, that points that the lack of a portal that accommodates OER for Arts Graphics is one of the greatest obstacles to the use of OER. One of the guidelines recently established by UNESCO (2024) is in line with this need, as it promotes supporting and maintaining peer networks to share OER in different subjects and languages.

In any case, DECriS LIS institutions also highlighted some advantages of using OER, both from the point of view of academics and students. Faculty perceived OER as being easy to reuse, adapt, and access quickly. These are, in fact, some of the best-known benefits of OER, as CC licenses (depending on the type) give permission to modify the content to create new OER that are culturally relevant and inclusive for different contexts and learners ( Van Allen & Katz, 2020). Students also appreciated OER, due to the immediacy and usefulness of digital materials (such as tutorials) and their availability outside the classroom. Likewise, Cheung *et al*. (2022) found that students generally consider OER useful as supplementary course materials or to help them to understand concepts. Angelopoulou *et al*. (2022), who studied student feedback on the use of open textbooks, concluded that students with higher motivation to learn perceived this kind of OER as a better option than the traditional textbook in comparison to less motivated students.

It should be noted that students in our research seem to understand OER more as digital resources available online rather than specific open learning material. The study of Arcebuche (2022) also identified that students associated OER with videos, images, e-books and other online resources that they could usually access through webpages, wikis, web engines and YouTube.

Regarding the creation of OER, DECriS LIS institutions do not exhibit much evidence either. Those faculty who produced them did it in the form of video, project-based materials and gamification resources. Nevertheless, these resources were often not conceived as OER; that is, they did not have the key characteristics of assigning open licenses, or sharing them openly by depositing them into a repository, etc. Therefore, some of the new resources created seem to be more innovation educational materials rather than OER. These results are also consistent with the findings of other authors ( Biernat *et al*., 2022; Kurt, 2019; Sunar *et al*., 2022), which indicates that academics are confused about open licenses, and that they use and create OER under misconceptions or without knowing the theoretical concept about them. On the other hand, there are other studies about OER in the times of COVID-19 that show the creation of some instructional videos designed specifically as OER; in the sense that they are open for reuse and enable student engagement and accessibility ( Boté-Vericad & Miguillón, 2012; Doi *et al*., 2022).

All these practices of OER use and creation identified among LIS academics and students evidence a limited readiness for OER implementation during the COVID-19 pandemic. The European Commission (2020) also identified this aspect in a survey among different HEIs on how they could have been better prepared to face the pandemic. Alhaj *et al*. (2021), also reported that the unpreparedness of institutions and their teaching staff has led in many cases (in North America and Europe) to poor quality ERT rather than well-constructed online learning. Similar results were informed in Turkey ( Sunar *et al*., 2022), where HEIs were found to be unprepared for online education, with faculty and students lacking the necessary digital skills.

In our study, LIS institutions have mainly focused on tools and workspaces for distance education but have not explored the further possibilities offered by digital OER. The online environment has helped to innovate education and to develop some project-based learning. However, since it is needed a certain shift in mindset and an investment of time for designing and implementing OER, the ERT period caused by the COVID-19 pandemic does not seem to have been the most appropriate time for doing this. Our faculty reported that e-learning and teaching require working on a more carefully constructed and detailed teaching plan and resources. Therefore, for those institutions that were transitioning to online education and becoming familiar with OER for the first time, it seems that the pandemic was not a good time to create new and purposely designed resources from scratch.

This overall scenario shows that the pandemic has been a catalyst for digital transformation in HEIs around the world. The online environment has helped to innovate education and to develop some project-based learning. However, the ERT period caused by the COVID-19 pandemic does not seem to have been the most appropriate time for doing this. According to Alhaj *et al*. (2021), the unpreparedness of institutions and their teaching staff has led in many cases (in North America and Europe) to poor quality ERT rather than well-constructed online learning. 

However, there were diverse contexts and experiences depending on the inequalities and different levels or speeds that have been observed in some countries and educational institutions. While some HEIs are in their infancy stage regarding awareness and engagement with OER and OEP, as we have observed in DECriS LIS institutions, other HEIs have an established culture of OER that has been further strengthened with the COVID-19 educational response. Some of the drivers have been the development of technological infrastructure as well as policies and strategies for a greater engagement with OE ( Bozkurt *et al*., 2020; Ossiannilsson *et al*., 2020; Nagashima & Hrach, 2021).

In this regard, the UNESCO (2019) Recommendation on OER also echoes the need to improve some of the aspects mentioned above through its areas of action, such as those of building capacity of stakeholders (i), developing supportive policies (ii) or encouraging the creation of inclusive and equitable quality OER (iii).

## Conclusions

### a) The impact of OER during COVID on the LIS HEIs analyzed in our research has not been strong, as they are in an early stage of OER implementation

In this study, we have seen that there are different paces of OER implementation in HEIs around the world. In the case of the LIS institutions in our research, they are mainly in a first stage of the process of adopting OER, which, according to Kaminski’s (2011) theory of diffusion of innovations, would correspond to the “Knowledge or Awareness” level. As a result, there would still be several implementation stages to go through before shifting from early awareness to full-fledged OER adoption.

This point is especially relevant in the case of the LIS discipline, since the scope of knowledge of LIS faculty is directly related to OER, in terms of their mission of providing access to information for all. Therefore, OER-specific programmes and open educational practice could be introduced into the LIS teaching, which would benefit faculty and students as well as set an example for other disciplines and university communities.

It needs to be understood that we are talking about OER considered as a well-defined typology of educational resource with some distinctive characteristics ( Miao *et al.*, 2019, p. 9; UNESCO, 2019) that differentiate them from the multitude of resources freely available on the Internet that can have a didactic application. As an example of those resources not considered OER, it can be said that a good number of teachers reported the recurrent use of YouTube and some other video portals to obtain resources to support their teaching. While some videos on YouTube may be the result of projects consciously carried out as OER in their canonical definition, and may be shared with the corresponding use notes and educational scope, the large majority of YouTube videos used would not correspond, in either the type of licence or the type of pedagogical contextualization, to a good praxis in the creation of OER. 

### b) OER have been used and created in different ways during the COVID-19 pandemic, depending on the level of awareness (Q1)

In general, there is little awareness about the role and impact that OER can have in education, especially in crisis situations. There was interesting evidence of educational resources being produced and made openly available, although not under a fully canonical model of what is meant by OER, for instance their incorporation into an OER repository or directory. Faculty endorse the OER philosophy, but their productions are not conceived as such under this label and with these characteristics. LIS faculty were not very familiar with OER in terms of how to create them or where to find them, nor with the use of the open licences. However, they showed a good attitude towards using some OER as additional learning resources and towards the innovative creation of more interactive materials. As some OER solutions that faculty used during the pandemic as an "experimental approach" have worked well, now would be the time to develop a consistent teacher training programme.

From the students' perspective, it seems that OER will continue to evolve as an important source of learning materials, as students were especially pleased with the accessibility and reusability of the resources, and they recognized the benefits of using complementary and specialized resources. Although students were not particularly aware of OER during the pandemic, they seem now to recognize their usefulness more than before. Students did not have the perception that they had used OER provided by teachers. When they did talk about that, their knowledge was blurred between open access academic papers and books, OER and free resources of any kind on the Internet. As future librarians and as information science specialists, and therefore as a potential OER advocates, it is a bit disappointing to see such low awareness about OER. This is indicative of the necessity to include the issue of OER in LIS educational programmes.

### c) The barriers and advantages of OER reported during COVID-19 are consistent with other previous literature (Q2)

This study, situated in the specific field of LIS, mirrors the results of OER adoption studies in other fields. In any case, this research emphasizes two main aspects: the lack of knowledge about OER and their characteristics and the findability problem with OER scattered across different sources, which are mostly unknown to users. Since being familiar with OER positively affects attitudes towards them, faculty who are unaware of OER are likely to be slow to adopting them and will use OER for supplemental purposes in their courses. Moreover, the fact that many faculty used YouTube as a key source for locating videos to include in their lessons, demonstrates the importance of effective discovery mechanisms and the need for more training in OER and specialized sources.

### d) Some of the main lessons learned that will help to foster OER post-pandemic (Q3) are the need to learn new skills, have more training and have more collaboration and institutional support

Capacity building in OER, as well as know-how to create them and relevant sources to share them, are necessary for them to become generalized. In addition, more collaboration and institutional support are required, both in terms of centres and departments involved (faculty, libraries, educational innovation, etc.), as well as strategies and rewards to foster OER.

Regarding interdepartmental collaboration, LIS faculty could create and employ OER more widely by relying on and working together with their academic libraries, which often stand out by leading or supporting the OER movement. As for the contribution of libraries, it can range from providing library materials for OER creation, selecting existing OER for use, and providing infrastructure (repositories and other platforms), to involving library staff directly in OER development and academic support. In this regard, LIS discipline could be helpful in OER context due to the experience of its professionals (academics and librarians) and their specialization in searchability, repositories management and the ability to identify thematic OER.

### Further directions

The overview and the statements about the low use of OER in this paper are the result of fieldwork with LIS faculty and students from five HEI in four countries (Germany, Bulgaria, Croatia and Spain). Therefore, it should be considered as a new partial contribution to the study of the OER implementation. This limitation implies that the findings reported cannot be generalized to a worldwide level or to any thematic area of university education. The implementation and vitality of OER is certainly very varied, as there are different contexts in which different adoption speeds are observed, and in which the results would certainly be more positive. There are some geographies, disciplines and institutions that are much more advanced in terms of OER, but definitely others are at a similar stage to the one described in this paper, or even worse.

So that, further investigation is clearly needed to complete the results provided by the present study. One of the possible directions would be to include LIS faculties from other European universities. It is also suggested that a future study should consider other factors, such as issues related to OER infrastructure or the career-related incentives for the creators of OER, to improve the educational experience of both teachers and students, and development of new policies and regulations affecting education, beyond those devised in response to unforeseen emergencies disrupting education.

In order to consolidate the results on evaluation of OER use with a global perspective, it would also be very appropriate and necessary to conduct a systematic literature review of works similar to ours, which would shed light on the factors that explain the barriers to OER creation, sharing and use. Finally, in line with the current rise of artificial intelligence (AI), it would also be interesting to study the intersection of OER and AI, such as the impact of emerging technologies on LIS area.

## Ethics and consent

In compliance with the COREQ guidelines for interviews and focus groups, our research prioritized ethical considerations. Written informed consent was diligently obtained, ensuring participants understand the study's purpose. Stricter measures, including interviewees quotations anonymization at the results outputs and secure data storage, were implemented to guarantee confidentiality. The design of the study, as well as the process of data collection and its safekeeping, was approved, on 2021-11-05 by the Bioethics Commission of the University of Barcelona (CBUB) [Institutional Review Board (IRB00003099) http://www.ub.edu/comissiobioetica/en], as the Barcelona team led the IO-2 work package of the DECriS Project.

## Data Availability

Due to the sensitive nature of the research and the legal restrictions agreed with the Bioethics Commission of University of Barcelona-CBUB [Institutional Review Board (IRB00003099) http://www.ub.edu/comissiobioetica/en] the supporting data (audio recordings and transcriptions) is not available. The consent of the participants in the interviews and focus groups was obtained under this condition. However, a fairly complete selection of anonymised excerpts from interviews and focus groups is available as quotations embedded at the complete analysis of the responses that can be found at the Intellectual Output-2 report of the DECriS Project, uploaded to the institutional repository of the University of Barcelona: http://hdl.handle.net/2445/201761 (This report is a working paper and was not peer reviewed). Figshare: DECriS Project Intellectual Output 2: Qualitative Research Instruments. https://doi.org/10.6084/m9.figshare.25541524.v1 This project contains the following extended data: Guide for teachers’ interviews Students’ focus groups moderator guide Data are available under the terms of the Creative Commons Attribution 4.0 International license (CC-BY 4.0).
