# Supplementary material for: Assessment of the use of Open Educational Resources at five European Library and Information Science higher education institutions during and post-COVID-19 pandemic
Source: Open Res Eur. 2024 Oct 25;4:95. Originally published 2024 Apr 25. [Version 3] doi: 10.12688/openreseurope.17457.3 (PMC11523556; doi:10.12688/openreseurope.17457.3)
Supplement: Peer review history

# Reviewer report: Reviewer response for version 3

**DOI:** 10.21956/openreseurope.20312.r45508

**Published:** 2024-11-05

**Authors:** Som Nath Ghimire

**Affiliations:** 1 Nepal Open University, Lalitpur, Nepal

Dear Authors,

 Thank you for your attention to my previous comments and other reviewers' concerns. With this, I have no further concerns regarding your paper. However, I suggest that having your paper copyedited by a professional copyeditor would greatly improve its clarity and overall rigor. Congratulations and best wishes.

If applicable, is the statistical analysis and its interpretation appropriate?

Not applicable

Is the study design appropriate and is the work technically sound?

Partly

Is the work clearly and accurately presented and does it engage with the current literature?

Partly

Are the conclusions drawn adequately supported by the results?

Partly

Are sufficient details of methods and analysis provided to allow replication by others?

Partly

Are all the source data and materials underlying the results available?

Partly

Reviewer Expertise:

Higher education; technology in education; online and distance education; integrity; academic dishonesty; surveillance; discourse studies; gender studies

I confirm that I have read this submission and believe that I have an appropriate level of expertise to confirm that it is of an acceptable scientific standard.

# Reviewer report: Reviewer response for version 3

**DOI:** 10.21956/openreseurope.20312.r45507

**Published:** 2024-10-28

**Authors:** Fahriye Altinay

**Affiliations:** 1 Near East University, Northern Cyprus, Turkey

This is a good study to give insights on OER. I suggest authors to elaborate the following statement in below that is stated parts of discussion.

 `At the beginning of our study, we had assumed that, in crisis situations, OER would be a key resource for online ERT. But all indications are that this assumption as a starting point was not correct. That is, if OER were not even minimally present in the mental framework or current routines of teachers in normal situations before COVID-19 (as direct users, recyclers or creators), OER was unlikely to be considered in crisis situations.`

 There should be strong argumentation by updated references how authors come up with this paragraph.

 In addition to this, reasons of choosing this topic as the need for the study need to be addressed.

If applicable, is the statistical analysis and its interpretation appropriate?

Not applicable

Is the study design appropriate and is the work technically sound?

Partly

Is the work clearly and accurately presented and does it engage with the current literature?

Partly

Are the conclusions drawn adequately supported by the results?

Partly

Are sufficient details of methods and analysis provided to allow replication by others?

Yes

Are all the source data and materials underlying the results available?

Partly

Reviewer Expertise:

online education, management, disability, strategic management, educational technology and quality

I confirm that I have read this submission and believe that I have an appropriate level of expertise to confirm that it is of an acceptable scientific standard.

# Reviewer report: Reviewer response for version 2

**DOI:** 10.21956/openreseurope.19979.r44263

**Published:** 2024-10-09

**Authors:** Michelle Harrison

**Affiliations:** 1 Thompson Rivers University, Kamloops, British Columbia, Canada

Overall, this study provides insight into OER adoption during ERT and highlights the needs for institutional support and guidance to improve awareness and help build the skills for faculty to adopt and adapt OER to their local context. The study provides additional insights into faculty/student use, and provides practical suggestions, consistent with the current literature, on how to overcome barriers to adoption and creation of OER in a global context. I have a few suggestions below on how to improve the clarity of the discussion and methodology sections. Just a note that there are some typos/grammatical errors throughout, and a repeated paragraph in the literature review (starting with “A systematic review by Otto et al).

**Study design**

 Methodology section: I was looking for better alignment between the chosen qualitative approach and a theoretical framework. Content analysis is a qualitative method (not framework for analysis) so a few sentences in this section would be helpful. Interviews and focus groups are data collection techniques (I don’t believe considered methodologies) – so these this needs to be clarified.

 It was not clear how you came up with your general coding scheme – did the themes emerge through inductive coding, or were these overall themes identified previous to coding? Earlier you state you used inductive coding, but it was not clear based on your final paragraph in the methods section (it states that there were six thematic sections in the questionnaire). Perhaps add a sentence or two to clarify your approach.

**Discussion**

 The discussion was not always clear. For example in the second paragraph I think your overall point was that across all five institutions, one factor, digital literacy (digital and computing competency), likely determined OER adoption/uptake, and that this uptake was generally quite low. This was not clearly stated, so a bit of rewording could strengthen your arguments.  

 Barriers to OER paragraph – provide a bit more detail about “legal uncertainty” – is this related to copyright? You don’t mention legal uncertainty as a barrier within your results section – so perhaps unpack that more here to align with your findings (if appropriate).

 “One of the guidelines recently established by UNESCO (2024) is in line with this need, as it promotes supporting and maintaining peer networks to share OER in different subjects and languages.” – briefly outline what this guideline states for your readers.

 Early in your discussion you indicate that OER uptake was low, and later indicate that there was limited readiness…but in the advantages section, you highlight how faculty and students indicate they are easy to reuse, adapt and access – can you add a bit of discussion about these contradictory overall results (perhaps highlight this in your discussion and/or conclusion sections).

 There were a few typos (first paragraph “curses” should be “cases”).

 You could also refer to some of the institutional adoption of OER studies to help ground some of your recommendations in your conclusion.[1,2]

https://www.ecampusontario.ca/wp-content/uploads/2019/08/2019-08-07-skimore-oe-policy-report.pdf

If applicable, is the statistical analysis and its interpretation appropriate?

Not applicable

Is the study design appropriate and is the work technically sound?

Yes

Is the work clearly and accurately presented and does it engage with the current literature?

Yes

Are the conclusions drawn adequately supported by the results?

Partly

Are sufficient details of methods and analysis provided to allow replication by others?

Partly

Are all the source data and materials underlying the results available?

Yes

Reviewer Expertise:

Open educational practices, learning design, qualitative

I confirm that I have read this submission and believe that I have an appropriate level of expertise to confirm that it is of an acceptable scientific standard, however I have significant reservations, as outlined above.

## References

[1] : Institutional Culture and OER Policy: How Structure, Culture, and Agency Mediate OER Policy Potential in South African Universities. *The International Review of Research in Open and Distributed Learning* .2016;17(5) : 10.19173/irrodl.v17i5.2523 10.19173/irrodl.v17i5.2523

[2] : Heterogeneity of Genetic Damage in Cervical Nuclei and Lymphocytes in Women with Different Levels of Dysplasia and Cancer-Associated Risk Factors. *Biomed Res Int* .2015;2015: 10.1155/2015/293408 293408 10.1155/2015/293408 26339603 PMC4538336

# Response

**Published:** 2024-10-16

**Authors:** Cristóbal Urbano

**Affiliations:** Departament de Biblioteconomia, Documentació i Com, Universitat de Barcelona, Barcelona, Catalonia, Spain

**Comment #0:** *Overall, this study provides insight into OER adoption during ERT and highlights the needs for institutional support and guidance to improve awareness and help build the skills for faculty to adopt and adapt OER to their local context. The study provides additional insights into faculty/student use, and provides practical suggestions, consistent with the current literature, on how to overcome barriers to adoption and creation of OER in a global context. I have a few suggestions below on how to improve the clarity of the discussion and methodology sections. Just a note that there are some typos/grammatical errors throughout, and a repeated paragraph in the literature review (starting with “A systematic review by Otto et al).*

  

- RE: We are grateful for all the comments of reviewer Michelle Harrison, which help us to positively assess the relevance of the results obtained, while at the same time helping us to introduce some improvements or clarifications. The response to each of them is detailed below:

**Comment #1: *Study design.*** *Methodology section: I was looking for better alignment between the chosen qualitative approach and a theoretical framework. Content analysis is a qualitative method (not framework for analysis) so a few sentences in this section would be helpful. Interviews and focus groups are data collection techniques (I don’t believe considered methodologies) – so these this needs to be clarified. *

- RE: Our study has been planned as a case study to obtain empirical evidence of the OER’s use, and to gather experiences and opinions on the lessons learned. It could be said that our work is “deliberately agnostic” when it comes to theoretical frameworks on education. Further works devoted to theoretical studies could benefit of our empirical data. So that, we have corrected the denomination of Content analysis as “theoretical framework” to “qualitative method”; also, we changed the name of the section to “Materials and methods”.

 
*It was not clear how you came up with your general coding scheme – did the themes emerge through inductive coding, or were these overall themes identified previous to coding? Earlier you state you used inductive coding, but it was not clear based on your final paragraph in the methods section (it states that there were six thematic sections in the questionnaire). Perhaps add a sentence or two to clarify your approach.*

  

- RE: Definitely, the specific themes (identified in Table 2 as Subcode) emerge through inductive coding. But all subcodes were nested on the go of coding inside 4 general themes linked to the areas where we have figured out our study could reach results: Adaptations, Problems, Advantages, Improvements/Lessons learned. We have rephrased the final paragraph.

**Comment #2: *Discussion.*** *The discussion was not always clear. For example in the second paragraph I think your overall point was that across all five institutions, one factor, digital literacy (digital and computing competency), likely determined OER adoption/uptake, and that this uptake was generally quite low. This was not clearly stated, so a bit of rewording could strengthen your arguments.  *

- RE: We have changed the order of the sentences at the second paragraph, and we have changed the text to highlight the idea that “that teachers' previous experience with OER and the use of digital media in subjects more suited to them, constitutes a key differential factor in the level of use of OER during ERT.”

*Barriers to OER paragraph – provide a bit more detail about “legal uncertainty” – is this related to copyright? You don’t mention legal uncertainty as a barrier within your results section – so perhaps unpack that more here to align with your findings (if appropriate).*

- RE: Yes, it’s about how to use any digital resource from the Net when the resources don’t have clear CC licences. The link with Results sections is in the quote of one of the teachers [OJ_6] that said: “I had problems finding some of the materials and resources that I could use […] I think we should have more repositories of OER and different digital materials that are available to use, so there are more in the public domain or licenced through Open Licencing by CC. And I think we should be more open to sharing resources. We talk about open science and open education, but in practice we don't see it so much.” To complement that quotation, we have added a sentence to present the issue of legal uncertainty.

*“One of the guidelines recently established by* *UNESCO (2024)* *is in line with this need, as it promotes supporting and maintaining peer networks to share OER in different subjects and languages.” – briefly outline what this guideline states for your readers.*

- RE: We have added a sentence and a citation to state what does it mean for our readers, especially those in the LIS area.

*Early in your discussion you indicate that OER uptake was low, and later indicate that there was limited readiness…but in the advantages section, you highlight how faculty and students indicate they are easy to reuse, adapt and access – can you add a bit of discussion about these contradictory overall results (perhaps highlight this in your discussion and/or conclusion sections).*

  

- RE: Yes, teachers’ statements are contradictory. From the ideal and theoretical perspective, they praise the OER possibilities and advantages, but their actual experience and behaviour does not match their mostly theoretical thoughts about advantages. We have added a sentence to stress that contradiction.

*There were a few typos (first paragraph “curses” should be “cases”).*

  

- RE: Thank you. We have revised throughout the paper to check for typos, but in the case of the first paragraph, we keep “courses” as the sentence is about the process of adaption of the “courses” of the educational programmes when the pandemic broke out

   
**Comment #3: *Conclusion.*** *You could also refer to some of the institutional adoption of OER studies to help ground some of your recommendations in your conclusion.[1,2]*

*https://www.ecampusontario.ca/wp-content/uploads/2019/08/2019-08-07-skimore-oe-policy-report.pdf* *. *References: 1. Cox G, Trotter H: Institutional Culture and OER Policy: How Structure, Culture, and Agency Mediate OER Policy Potential in South African Universities. The International Review of Research in Open and Distributed Learning. 2016; 17 (5): | Publisher Full Text / 2. Alvarez-Moya C, Reynoso-Silva M, Canales-Aguirre AA, et al.: Heterogeneity of Genetic Damage in Cervical Nuclei and Lymphocytes in Women with Different Levels of Dysplasia and Cancer-Associated Risk Factors. Biomed Res Int. 2015; 2015 293408 PubMed Abstract | Publisher Full Text  

- RE: We believe that in the scope of the paper we have written, it does not make sense to include these references in the conclusion section. Indirectly, however, the suggestion that follows from the reviewer's comment is integrated in the ‘Further directions’ section of the paper. We have modified the wording on the conduct of a systematic literature review to include more explicitly works on institutional adoption of OER studies.

# Reviewer report: Reviewer response for version 2

**DOI:** 10.21956/openreseurope.19979.r44258

**Published:** 2024-09-25

**Authors:** Lancelord Siphamandla Mncube

**Affiliations:** 1 University of South Africa, Pretoria, South Africa

**Keywords**

 Some of the keywords, such as Emergency Remote Teaching (ERT) and Digital education are just imposed, and nothing is said about them in the text.

**Introduction**

 The introduction needs to be captivating and focus on a substantiation of the chosen research interest, focus or problem.

 The researchers need to identify a gap in existing research about the research interest, focus or problem in the study, as implied in the title. Studying “Assessment of the use of Open Educational Resources at five European Library and Information Science”- you need to put a reader on a clear picture of what studies have been conducted in LIS context based on OER. In so doing you will be in the position to identify existing gaps.  

 Researchers may succeed in substantiating the existence of the chosen research problem (i.e. the existing gap in research) through valid evidence obtained from recent literature.

 Briefly outline of the problem exposing the reader to the problem you want to investigate. Here you deliberate on the phenomenon under investigation looking at what the literature says about it. In this section, you need to provide a global picture of what is happening under the topic that is being studied. For example, when writing about OER in LIS sectors ; you need to provide a discussion globally for example, choose at least 4 countries from the West including Asia (UeSA, UK, China, Africa, Brazil,  India etc.). Thereafter discuss what is happening at the national levels (countries) including locally (the area where your study will be conducted).

**Statement of the problem**

 At this stage, the study research problem is not well articulated. With the introduction and background as basis, discuss the research problem in no more than four full sentences. Make sure that the research problem directly links with the information contained in the preceding introduction and background. Convince readers that what you intend to investigate is a real problem and is worth researching.  

**Significance/value of the study**

 The OER topic is often associated with the field of Education. Therefore you need to create a strong justification on how OER potential in the field of LIS. Briefly discuss the value that the proposed research will have for the study of science in general, the particular discipline(s) and/or field(s) of study within which the study is demarcated, as well as its value for relevant stakeholders (as applicable). In a nutshell, will this study make any contribution to national or international debates? Will it make any contribution in terms of theory (theory underpinning your study)?/impact within the disciplinary/interdisciplinary context; any contribution in stimulation further research?

**Consider infusing Theoretical Frameworks underpinning the study.**

 Place the proposed research within an appropriate theoretical framework that will guide the study. What theory or theories underpin your study? (Provide relevant literature – in this case, you can use any relevant literature regardless of the date). Limit the number of theories to be used but dig dipper into the theories you have opted to use. You need this theory as you are formulating the research questions. At this stage is difficult to understand how your research question were formulated. You need the theoretical framework or conceptual framework when you are reviewing literature. In this paper, justice is not done concerning the review of literature.  You will need this theoretical framework when discussing your results or doing the writing up.

**Methodology**

 You need to articulate if ethical clearances were obtained from all involved institutions. Provide a summary of the measures that you will take to satisfy the generally accepted ethical requirements for research. How the following five aspects were addressed during the research process:

-  Issues of privacy,
- Consent,
- Permission to conduct research etc.
- Confidentiality
- Voluntary participation
- Informed consent etc.

**Findings**

 In this section, you provided a lot of quotations. You need to narrate your results. We want to hear your own voice when presenting results rather than imposing quotations. Presenting findings guided by the theoretical framework or conceptual framework always creates a better logic in this calibre of research.

**Conclusion**

 Reframe the inclusion of citations in this section.

If applicable, is the statistical analysis and its interpretation appropriate?

Not applicable

Is the study design appropriate and is the work technically sound?

Yes

Is the work clearly and accurately presented and does it engage with the current literature?

Partly

Are the conclusions drawn adequately supported by the results?

Partly

Are sufficient details of methods and analysis provided to allow replication by others?

Yes

Are all the source data and materials underlying the results available?

Yes

Reviewer Expertise:

NA

I confirm that I have read this submission and believe that I have an appropriate level of expertise to confirm that it is of an acceptable scientific standard, however I have significant reservations, as outlined above.

# Response

**Published:** 2024-10-16

**Authors:** Cristóbal Urbano

**Affiliations:** Departament de Biblioteconomia, Documentació i Com, Universitat de Barcelona, Barcelona, Catalonia, Spain

We welcome and appreciate all comments and suggestions for improvement from reviewer Lancelord Siphamandla Mncube. We consider some of them useful to introduce changes, while others are interesting, but we consider that the huge considerable changes associated with them will trigger a very different paper like a systematic review or a theoretical paper. It is with great pleasure that we upload a revised version 3 to cope with all the reviewers. --------------------------------------------------------------------------------- The response to each comment is detailed below:

**Comment #1: *Keywords.*** *Some of the keywords, such as Emergency Remote Teaching (ERT) and Digital education are just imposed, and nothing is said about them in the text.*

**Response:** We have dropped the keyword “Digital education”, but we keep the keyword “Emergency Remote Teaching (ERT)”, as it is included in the section “OER global initiatives” at the “Introduction”.  We have added more context and a citation to a flagship reference focused on that concept.

**Comment #2: *Introduction.*** *The introduction needs to be captivating and focus on a substantiation of the chosen research interest, focus or problem. The researchers need to identify a gap in existing research about the research interest, focus or problem in the study, as implied in the title. Studying “Assessment of the use of Open Educational Resources at five European Library and Information Science”- you need to put a reader on a clear picture of what studies have been conducted in LIS context based on OER. In so doing you will be in the position to identify existing gaps.  Researchers may succeed in substantiating the existence of the chosen research problem (i.e. the existing gap in research) through valid evidence obtained from recent literature. Briefly outline of the problem exposing the reader to the problem you want to investigate. Here you deliberate on the phenomenon under investigation looking at what the literature says about it. In this section, you need to provide a global picture of what is happening under the topic that is being studied. For example, when writing about OER in LIS sectors ; you need to provide a discussion globally for example, choose at least 4 countries from the West including Asia (UeSA, UK, China, Africa, Brazil,  India etc.). Thereafter discuss what is happening at the national levels (countries) including locally (the area where your study will be conducted).*

**Response: **Some improvements have been made in the section “Main actors, context and scenarios of OER” to reinforce our view, expressed in that section, that the literature about OER use is uneven and with little-investigated empirical effects of use of OER (see Otto et al, 2021). Furthermore, it has to be considered that our work contributes to the literature with a case study not previously done about use of OER during the pandemic at LIS European institutions, through qualitative fieldwork using surveys and focus groups. It is not intended to be an exhaustive state of the art in the form of a literature review, as that would be another type of article. We believe that the Introduction builds on a sufficient body of previous work to show that there are few studies that have carried out concrete evaluations of the use of OER in the university environment, considering the actual opinions of teachers and students. The literature presented in the Introduction is complemented by that used in the Discussion of the results sections. In total, 54 references have been analysed, most of them specifically linked to the field of OER in university education during the pandemic. We think most of the things requested in this comment by the reviewer can be traced in our text devoted to discussing the results, and also in the Introduction.

**Comment #3: *Statement of the problem.*** *At this stage, the study research problem is not well articulated. With the introduction and background as basis, discuss the research problem in no more than four full sentences. Make sure that the research problem directly links with the information contained in the preceding introduction and background. Convince readers that what you intend to investigate is a real problem and is worth researching.*

**Response: **We have added new text to the section “Purpose and research questions” to give a more detailed idea of the statement of the problem.

**Comment #4: *Significance/value of the study.*** *The OER topic is often associated with the field of Education. Therefore you need to create a strong justification on how OER potential in the field of LIS. Briefly discuss the value that the proposed research will have for the study of science in general, the particular discipline(s) and/or field(s) of study within which the study is demarcated, as well as its value for relevant stakeholders (as applicable). In a nutshell, will this study make any contribution to national or international debates? Will it make any contribution in terms of theory (theory underpinning your study)?/impact within the disciplinary/interdisciplinary context; any contribution in stimulation further research?* **Response: **​​​​​​​We have added new text to the section “Purpose and research questions” to give a more detailed idea of the statement of the problem and the significance of the takeaways of the research outputs.

**Comment #5: *Consider infusing Theoretical Frameworks underpinning the study.*** *Place the proposed research within an appropriate theoretical framework that will guide the study. What theory or theories underpin your study? (Provide relevant literature – in this case, you can use any relevant literature regardless of the date). Limit the number of theories to be used but dig dipper into the theories you have opted to use. You need this theory as you are formulating the research questions. At this stage is difficult to understand how your research question were formulated. You need the theoretical framework or conceptual framework when you are reviewing literature. In this paper, justice is not done concerning the review of literature.  You will need this theoretical framework when discussing your results or doing the writing up.*

**​​​​​​​Response: **​​​​​​​ We think that infusing theoretical frameworks underpinning the study is beyond the purpose of our study, planned as a case study to obtain empirical evidence of the OER’s use, and to gather experiences and opinions on the lessons learned. It could be said that our work is “deliberately agnostic” when it comes to theoretical frameworks on education. Further works devoted to theoretical studies could benefit of our empirical data.

**Comment #6: *Methodology.*** *You need to articulate if ethical clearances were obtained from all involved institutions. Provide a summary of the measures that you will take to satisfy the generally accepted ethical requirements for research. How the following five aspects were addressed during the research process:  Issues of privacy / Consent / Permission to conduct research etc. / Confidentiality / Voluntary participation / Informed consent etc.* **​​​​​​​Response: **Before the publication of the preprint version, Open Research Europe (ORE) editorial managers checked all the requirements for the manuscript, to which the reviewer refers in this comment. If the manuscript has finally been disseminated in ORE, it is because the requirements have been met. Detailed information on this can be found in the ‘Methodology’ section of the paper (now renamed as “Materials and methods”).

**​​​​​​​Response: **​​​​​​​ Moreover, the key information about these requirements is repeated in the statement under the “Ethics and consent” section at the end of the paper: “In compliance with the COREQ guidelines for interviews and focus groups, our research prioritized ethical considerations. Written informed consent was diligently obtained, ensuring participants understand the study’s purpose. Stricter measures, including interviewees quotations anonymization at the results outputs and secure data storage, were implemented to guarantee confidentiality. The design of the study, as well as the process of data collection and its safekeeping, was approved, on 2021-11-05 by the Bioethics Commission of the University of Barcelona (CBUB) [Institutional Review Board (IRB00003099) http://www.ub.edu/comissiobioetica/en], as the Barcelona team led the IO-2 work package of the DECriS Project.”

**Comment #7: *Findings.*** *In this section, you provided a lot of quotations. You need to narrate your results. We want to hear your own voice when presenting results rather than imposing quotations. Presenting findings guided by the theoretical framework or conceptual framework always creates a better logic in this calibre of research.*

**​​​Response: **: The outputs of our work, for the sake of the clarity of our text, are presented in two consecutive and related sections. First, “Results” as a reasoned inventory of the main situations and experiences with OER derived from actual quotations captured at the interviews and focus groups with the participants. Every set of quotations is framed with a narrative comment and a headline of the issue/idea involved. Second, in the “Discussion” section we go deeper in the presentation of finding with our voice, but with the contrast with other works from the literature on the issue. In the “Results” section, we followed our agnostic and inductive approach to read and interpret the transcriptions to give the voice to the participants. In the “Discussion” section, it was the time for us as researchers to use our own voice to present results.

**Comment #8: *Conclusion.*** *Reframe the inclusion of citations in this section.*

**​​​​​​​​​​Response: ** We believe that the very few citations that appear (only 3) in this section are necessary for a correct understanding of what is mentioned in the paragraphs in which they are present.

# Reviewer report: Reviewer response for version 2

**DOI:** 10.21956/openreseurope.19979.r44073

**Published:** 2024-09-17

**Authors:** Som Nath Ghimire

**Affiliations:** 1 Nepal Open University, Lalitpur, Nepal

**Comments to the authors**

 Thank you for substantially revising the earlier version of your paper to address my previous comments. The improvements you have made have now significantly strengthened the clarity and readability of the paper and its contribution to the field under research scrutiny.

1. The revised title now aligns well with your research objectives and reflects the study's data and scope.
2. The introduction and literature review sections have been significantly improved with recent publications, providing a solid foundation for your study. These updates have provided a well-furnished background, particularly concerning OER concepts and practices in higher education.
3. The research gap, study objectives, and key contributions are now clearly articulated. The revisions have added considerable depth to the paper's relevance in the research area under scrutiny.
4. Thank you for clarifying participant profiles in the methodology and analysis sections.
5. The critical interpretation of your findings in the discussion section has significantly improved the rigor of your line of reasoning vis-à-vis higher education students' and faculties’ perceptions and experiences of OER practices.
6. The inclusion of introductory paragraphs summarizing the authors’ observations and overall findings has now offered a clear overview of gaps in OER implementation and your paper’s contribution to OER practices in higher education.
7. Thank you for clarifying the scope of OER in the conclusion. Your distinction between the decline of "canonical" OER and the growing use of diverse open-access resources has both improved the relevance of your findings and offered a beautiful insight into the understanding of current trends of OER practices (which suggests the required revisions in canonical concepts of OER).
8. The addition of the "Further Directions" section has outlined future research projections and provides a clear pathway for continued scholarly conversations around OER concepts and practice.

Overall, the current version of the paper is much improved as a result of the above revisions made by the authors. However, I have observed that there are some minor slips in the paper (only a few of them are detailed at the end of this report since detailing each of them would be quite exhaustive), subject to correction. Along with these comments, I would also like to suggest that the authors get their paper sincerely copyedited to effectively convey the overall message of their work.

  

1. Please insert a period as it is missing at the end of the second paragraph of your theme **“** **OER global initiatives**”.
2. Please remove one of the following paragraphs as it appears twice under the theme **“** **Main actors, contexts and scenarios of OER”**: “ A systematic review by Otto *et al*. (2021) reported about the little-investigated empirical effects of the use of OER on established pedagogical approaches. Nevertheless, several studies have been identified on the faculty’s perspective regarding general awareness of OER ( Biernat *et al*., 2021; Christoforidou & Georgiadou, 2021; Watermeyer *et al*., 2021) and their attitudes to OER use ( Karataş *et al*., 2022; Otto, 2022; Sunar *et al*., 2022). Regarding the students’ view, apart from a few projects that focused on their perceptions of the usefulness of OER ( Arcebuche, 2022; Cheung *et al*., 2022), most have centred primarily on the students’ attitudes towards technological change in education ( Aguilera-Hermida, 2020; Händel *et al*., 2022).”
3.  Please adjust the following sentence in the literature that appears in the second paragraph of your theme **“** **Main actors, contexts and scenarios of OER” : **“ Otto *et al*. (2021) provided evidence that the research on OER had been, mainly, located in North America”
4. Period is missing at the end of the third paragraph of your theme **“** **Main actors, contexts and scenarios of OER”**
5. I suggest that the authors revise and reorganize the content in this section based on their theme since the contents in their current form seem a little mismatched and the arguments do not appear coherent.

Finally, I would like to congratulate the authors for their significant and valued contribution to existing knowledge about OER practices, their opportunities, and challenges in higher education.

If applicable, is the statistical analysis and its interpretation appropriate?

Not applicable

Is the study design appropriate and is the work technically sound?

Partly

Is the work clearly and accurately presented and does it engage with the current literature?

Partly

Are the conclusions drawn adequately supported by the results?

Partly

Are sufficient details of methods and analysis provided to allow replication by others?

Partly

Are all the source data and materials underlying the results available?

Partly

Reviewer Expertise:

Higher education; technology in education; online and distance education; integrity; academic dishonesty; surveillance; discourse studies; gender studies

I confirm that I have read this submission and believe that I have an appropriate level of expertise to confirm that it is of an acceptable scientific standard, however I have significant reservations, as outlined above.

# Response

**Published:** 2024-10-16

**Authors:** Cristóbal Urbano

**Affiliations:** Departament de Biblioteconomia, Documentació i Com, Universitat de Barcelona, Barcelona, Catalonia, Spain

We would like to thank reviewer Som Nath Ghimire for the helpful new comments and suggestions for the improvement of our manuscript version 2.  It is with great pleasure that we upload a revised version 3 to address new comments. The response to each of them is detailed below:

**Reviewer Comment #0:** *Thank you for substantially revising the earlier version of your paper to address my previous comments. The improvements you have made have now significantly strengthened the clarity and readability of the paper and its contribution to the field under research scrutiny. [...] Finally, I would like to congratulate the authors for their significant and valued contribution to existing knowledge about OER practices, their opportunities, and challenges in higher education.*

- **Author response: ** We appreciate the reviewer’s words acknowledging the appropriateness of the changes made in version 2 compared to version 1 to address their initial comments, alongside his congratulation to the significance of our contribution to the evidence-informed debate on the use of OER.

**Reviewer Comment #1:** *Please insert a period as it is missing at the end of the second paragraph of your theme **“OER global initiatives**”.*

- **Author response: **Done

**Reviewer** **Comment #2: ** Please remove one of the following paragraphs as it appears twice under the theme **“Main actors, contexts and scenarios of OER”**: “ A systematic review by Otto *et al*. (2021) reported about the little-investigated empirical effects of the use of OER on established pedagogical approaches. Nevertheless, several studies have been identified on the faculty’s perspective regarding general awareness of OER ( Biernat *et al*., 2021; Christoforidou & Georgiadou, 2021; Watermeyer *et al*., 2021) and their attitudes to OER use ( Karataş *et al*., 2022; Otto, 2022; Sunar *et al*., 2022). Regarding the students’ view, apart from a few projects that focused on their perceptions of the usefulness of OER ( Arcebuche, 2022; Cheung *et al*., 2022), most have centred primarily on the students’ attitudes towards technological change in education ( Aguilera-Hermida, 2020; Händel *et al*., 2022).”

- **Author response: **Done.

**Reviewer  Comment #3:** *Please adjust the following sentence in the literature that appears in the second paragraph of your theme **“Main actors, contexts and scenarios of OER” : **“* *Otto et al. (2021)* *provided evidence that the research on OER had been, mainly, located in North America”*

- **Author response: ** Adjusted with a new scope of the sentence, pointing out that research and debate mainly located in the USA is about Open textbooks.

**Reviewer Comment #4:** *Period is missing at the end of the third paragraph of your theme **“Main actors, contexts and scenarios of OER”***

- **Author response: **Done

**Reviewer** **Comment #5:** *I suggest that the authors revise and reorganize the content in this section based on their theme since the contents in their current form seem a little mismatched and the arguments do not appear coherent.*

- **Author response: **We have modified the first paragraph of the section ‘Main actors, contexts and scenarios of OER’ to give more consistency to the narrative that constitutes the thread of the section. Furthermore, we have moved the previous last paragraph to the next section “Purpose and research questions” as it helps to introduce what we have done in our study. Now, the actual last paragraph of the “Main actors...” section serves to close the literature contextualization we used to outline the purpose of our research.

# Reviewer report: Reviewer response for version 1

**DOI:** 10.21956/openreseurope.18866.r41596

**Published:** 2024-07-02

**Authors:** Som Nath Ghimire

**Affiliations:** 1 Nepal Open University, Lalitpur, Nepal

First of all, I would like to congratulate the authors for their contribution to the literature associated with Open Educational Resources (OER), which warrants a continued examination in higher education. I am particularly interested in reviewing this paper to know to what extent the COVID-19 pandemic prompted the wider understanding, creation, and implementation of Open Educational Resources in Library and Information Science (LIS) within higher education institutions in the countries under the study scrutiny. Having reviewed the paper, I suggest improvements in the following areas so that the rigor of the paper could be significantly strengthened.

1. Reconsider the title of the paper since the significant data collected and utilized are related to the faculty members' and students' narratives and reflections on OER implementation during the COVID-19 contexts although the respondents were interviewed during the post-pandemic situation.
2. Both the introduction and literature review sections would further benefit if the authors could substantially revise these sections with updated literature in the area under scrutiny.
3. In so doing, please review past and recent literature that principally agrees with the widely accepted definition of OER rather than solely relying on UNESCO's (2019) definition. Consider, if there are any disagreements regarding the concept of OER over the years.
4. The review section is poorly written and the line of reasoning for the current research is less than satisfactory. Please consider critically reporting the theoretical arguments and empirical research findings to set the broader context for the current research under scrutiny. This section could further benefit if the authors focus on reviewing literature in the areas associated with their research questions so that it would logically connect with the findings and discussion section.Â  Moreover, the literature section seems to have abruptly ended, which looks very odd. I suggest that the authors summarize the literature review in a short paragraph and show the research gap that warrants further examination.
5. In the methodology section, I wonder why the authors have not considered it important to give information about the participants, whether all of them belong to Library and Information Science or different departments. Unclarity about the details could further mislead the readers since the title of the paper and abstract are centered around this information. It is equally absent in the analysis section though the authors have mentioned this information in the discussion and conclusion section that the participants belonged to LIS. Â 
6. In the findings and analysis section, the paper could further benefit from a critical interpretation of data, which the paper hugely lacks in its current form. Also, I believe that the inverted commas in the quotes should be removed with the indentation the journal requires.
7. Equally important concerns for me are that since the research has been 'based on fieldwork carried out in the LIS departments of the universities of Barcelona (Spain), Hildesheim (Germany), Osijek and Zagreb (Croatia) and the University of Library Studies and Information Technologies in Sofia (Bulgaria)', there might be different experiences and perception of faculties and students toward OER based on their contexts. If authors have something interesting to share (probably not reported in the available literature as well as that has not been highlighted with clarity (compare and contrast) in the current study), I would encourage the authors to equally highlight these findings and discuss them. I believe that it would have a greater implication in the literature as well as guide future debates in the area.

If applicable, is the statistical analysis and its interpretation appropriate?

Not applicable

Is the study design appropriate and is the work technically sound?

Partly

Is the work clearly and accurately presented and does it engage with the current literature?

Partly

Are the conclusions drawn adequately supported by the results?

Partly

Are sufficient details of methods and analysis provided to allow replication by others?

Partly

Are all the source data and materials underlying the results available?

Partly

Reviewer Expertise:

Higher education; technology in education; online and distance education; integrity; academic dishonesty; surveillance; discourse studies; gender studies

I confirm that I have read this submission and believe that I have an appropriate level of expertise to confirm that it is of an acceptable scientific standard, however I have significant reservations, as outlined above.

# Response

**Published:** 2024-07-08

**Authors:** Cristóbal Urbano

**Affiliations:** Departament de Biblioteconomia, Documentació i Com, Universitat de Barcelona, Barcelona, Catalonia, Spain

We the authors greatly appreciate the reviewers' comments and time. Based on their suggestions, we will answer the reviewer’s observations and revise our paper over the next few weeks to submit a new, corrected version. Some of the team members involved in this paper will be on holiday for a few weeks, so it will take some time. Thank you for your patience. Sincerely, Cristóbal Urbano, corresponding author

# Response

**Published:** 2024-08-28

**Authors:** Cristóbal Urbano

**Affiliations:** Departament de Biblioteconomia, Documentació i Com, Universitat de Barcelona, Barcelona, Catalonia, Spain

We would like to thank reviewer 2 for the helpful comments and suggestions on our manuscript. It is with great pleasure that we upload a revised version. The response to each comment is detailed below, together with the changes we have made to version 2 of the paper where appropriate (new text can be seen through the tracked changes):

- **Comment #0:** *First of all, I would like to congratulate the authors for their contribution to the literature associated with Open Educational Resources (OER), which warrants a continued examination in higher education. I am particularly interested in reviewing this paper to know to what extent the COVID-19 pandemic prompted the wider understanding, creation, and implementation of Open Educational Resources in Library and Information Science (LIS) within higher education institutions in the countries under the study scrutiny. *

 
**RE:** We appreciate very much this general introductory comment, where the reviewer states the relevance of the issue of critical assessments on OER use during COVID-19 pandemic, that in our opinion has not received the attention it deserves in the literature on Emergency Remote Teaching (ERT) during the pandemic. OER are the subject of much theoretical and descriptive literature analysing their characteristics and potential, promoting its use from a decontextualized perspective without considering the barriers and challenges to its adoption, but assessment of their actual use in university education falls short of expectations. This is one of the most interesting and surprising evidences resulting from our study of the COVID-19 period, which strongly suggested that their actual use was low and unsystematic, even in what seemed to be a very favourable situation such as the ERT. Hence, the reflections that teachers and students expressed a posteriori on this situation are relevant for detecting barriers and conditioning factors on which to act in the future for a more profitable use of the OER, as well as a greater involvement of teachers in their creation.  

- **Comment #1**: *Reconsider the title of the paper since the significant data collected and utilized are related to the faculty members and students narratives and reflections on OER implementation during the COVID-19 contexts although the respondents were interviewed during the post-pandemic situation.*

 
**RE:** Since our study is related to both periods, COVID-19 (the focus of RQ 1 and RQ2) and Post COVID (RQ3) we have modified the paper title to clearly state we will deal with both periods. New title for version 2: Assessment of the use of Open Educational Resources at five European Library and Information Science higher education institutions **during and post COVID -19 pandemic**  

- **Comment #2:** *Both the introduction and literature review sections would further benefit if the authors could substantially revise these sections with updated literature in the area under scrutiny.*

**RE:** We have updated the literature by searching more recent publications on our main topics. We have identified six references that provide some new ideas or approaches, which we have added in our literature review and discussion sections.  

- **Comment #3:** *In so doing, please review past and recent literature that principally agrees with the widely accepted definition of OER rather than solely relying on UNESCO's (2019) definition. Consider, if there are any disagreements regarding the concept of OER over the years.*

**RE:** While there is no single definition of OER, the most recent UNESCO definition -included in the UNESCO OER Recommendation- is widely accepted: *“*OER are teaching, learning and research materials that make use of appropriate tools, such as open licensing, to permit their free reuse, continuous improvement and repurposing by others . . . *”* (Miao, Mishra, & Orr, 2019, p. 9). We have also reviewed some recent articles about OER and all of them used the same definition. That is why we have added it to the introduction. Also, we have linked the delineation of what defines an OER to a mention in the Conclusions Section,  contextualising the low use of OER among our study participants.  

- **Comment #4:** *The review section is poorly written and the line of reasoning for the current research is less than satisfactory. Please consider critically reporting the theoretical arguments and empirical research findings to set the broader context for the current research under scrutiny. This section could further benefit if the authors focus on reviewing literature in the areas associated with their research questions so that it would logically connect with the findings and discussion section.  Moreover, the literature section seems to have abruptly ended, which looks very odd. I suggest that the authors summarize the literature review in a short paragraph and show the research gap that warrants further examination.*

**RE:** We have modified the literature review section to clarify the line of reasoning of the current research. Firstly, as we discussed above, we have updated the literature review with some recent publications that we identified on our main research topics. We have also structured the section, covering from global OER implementation initiatives to the specificity and key/differential areas of our contribution, i.e. a case of 5 higher education institutions in the discipline of Library and Information Science and Europe (as thematic and spatial context) and the teaching staff and students (as main actors).  

- **Comment #5:** *In the methodology section, I wonder why the authors have not considered it important to give information about the participants, whether all of them belong to Library and Information Science or different departments. Unclarity about the details could further mislead the readers since the title of the paper and abstract are centered around this information. It is equally absent in the analysis section though the authors have mentioned this information in the discussion and conclusion section that the participants belonged to LIS.*

**RE:** We have clarified now that all interviewees (faculty and students) were from LIS Department/Faculty also in the Methodology and Analysis sections, as it has been suggested.  

- **Comment #6:** *In the findings and analysis section, the paper could further benefit from a critical interpretation of data, which the paper hugely lacks in its current form. Also, I believe that the inverted commas in the quotes should be removed with the indentation the journal requires.*

**RE:** Within the Discussion section, further critical interpretation of the data has been added, in order to connect the findings with other observed trends or recently emerging guidelines. As for the inverted commas, since we have followed all the editorial indications of ORE (and also performed at the final published versions by editing services from ORE), which has not indicated anything specific in this regard, we keep the text as it was - unless ORE expresses any new requirement.  

- **Comment #7:** *Equally important concerns for me are that since the research has been based on fieldwork carried out in the LIS departments of the universities of Barcelona (Spain), Hildesheim (Germany), Osijek and Zagreb (Croatia) and the University of Library Studies and Information Technologies in Sofia (Bulgaria), there might be different experiences and perception of faculties and students toward OER based on their contexts. If authors have something interesting to share (probably not reported in the available literature as well as that has not been highlighted with clarity (compare and contrast) in the current study), I would encourage the authors to equally highlight these findings and discuss them. I believe that it would have a greater implication in the literature as well as guide future debates in the area.*

**RE:** In general, as far as the use of OER is concerned, the situation in the institutions with similar disciplinary traditions had similar characteristics. It is worth noting the differential case of Hildesheim (Germany) whose more digital orientation and specialisation in postgraduate course would explain a more intense and consolidated use of OER before COVID-19. But in general, for similar academic subjects, we have not actually identified many different perceptions of faculty and students towards OER depending on their context. In any case, for all the five institutions, the more digital oriented was the academic subject or the teacher background, the more likely OER could have some presence during the ERT and beyond. We have noted this adding a new text at the beginning of the Discussion Section. 

# Reviewer report: Reviewer response for version 1

**DOI:** 10.21956/openreseurope.18866.r40378

**Published:** 2024-05-13

**Authors:** Fahriye Altinay

**Affiliations:** 1 Near East University, Northern Cyprus, Turkey

Paper`s title mentions the period of post covid 19 assessment but in the research questions first two of them are related to covid 19 period. Authors should state clear time period in relation to the focus of the study. 

 Significance of the study as a need for the study should be stated by updated references and it is essential to put importance to the key terms that makes this study different from others. 

 Results can be categorized based on research questions. Themes and codes can be created in the analysis in order to be more clarified in relation to the research focus.

 Future projections and suggestions should be added for both research areas and researchers.

If applicable, is the statistical analysis and its interpretation appropriate?

Not applicable

Is the study design appropriate and is the work technically sound?

Partly

Is the work clearly and accurately presented and does it engage with the current literature?

Partly

Are the conclusions drawn adequately supported by the results?

Partly

Are sufficient details of methods and analysis provided to allow replication by others?

Yes

Are all the source data and materials underlying the results available?

Partly

Reviewer Expertise:

online education, management, disability, strategic management, educational technology and quality

I confirm that I have read this submission and believe that I have an appropriate level of expertise to confirm that it is of an acceptable scientific standard, however I have significant reservations, as outlined above.

# Response

**Published:** 2024-07-08

**Authors:** Cristóbal Urbano

**Affiliations:** Departament de Biblioteconomia, Documentació i Com, Universitat de Barcelona, Barcelona, Catalonia, Spain

We the authors greatly appreciate the reviewers' comments and time. Based on their suggestions, we will answer the reviewer’s observations and revise our paper over the next few weeks to submit a new, corrected version. Some of the team members involved in this paper will be on holiday for a few weeks, so it will take some time. Thank you for your patience.  Sincerely, Cristóbal Urbano, corresponding author

# Response

**Published:** 2024-08-28

**Authors:** Cristóbal Urbano

**Affiliations:** Departament de Biblioteconomia, Documentació i Com, Universitat de Barcelona, Barcelona, Catalonia, Spain

We welcome all comments and suggestions for improvement from reviewer 1. We consider them useful, and it is with great pleasure that we upload a revised version. The response to each comment is detailed below, together with the changes we have made to version 2 of the paper where appropriate (new text can be seen through the tracked changes): **Comment #1:** *Papers title mentions the period of post covid 19 assessment but* *in the research questions first two of them are related to covid 19 period. Authors should state clear time period in relation to the focus of the study.* **RE:** Since our study is related to both periods, COVID-19 (the focus of RQ 1 and RQ2) and Post COVID-19 (RQ3), we have made a slight modification of the paper title to clearly state we will deal with both periods, like it is also stated in the abstract. New title for version 2:

- Assessment of the use of Open Educational Resources at five European Library and Information Science higher education institutions **during and post COVID -19 pandemic**

 
**Comment 2#:** *Significance of the study as a need for the study should be stated by* *updated references and it is essential to put importance to the key terms that makes this study different from others.*

**RE:**  We have updated references by searching new publications on OER use and LIS during COVID-19 period (and post-pandemic) at the European context on OER assessment, the key and differential focus of our study. We have identified a few articles, from 2023 and 2024, that provide some ideas or approaches which we have added in our literature review and discussion sections.  

**Comment 3#:** *Results can be categorized based on research questions* *. Themes and codes can be created in the analysis in order to be more clarified in relation to the research focus.* **RE:** The themes and codes presented in our study (and collected in Table II) are aligned with the general objectives of the DECriS project. Therefore, they are part of a broader study and have been designed from the same questionnaire script and from a first general analysis of the data, which considered a wide range of topics. For this reason, the coding was created in a general way and is not only categorized into OER research topics. As we consider that it is not appropriate to change the coding once the analysis is complete but, at the same time, we want to clarify the issue for readers (as the reviewer has pointed out) we have added a new paragraph in the Methodology section and we have adjusted Table II with tags in the first column to clarify. With this pair of changes, we aim to point out the connections between the general codes and our research themes and questions. We hope that this solution clarifies this issue.  

**Comment 4#:** *Future projections and suggestions should be added* *for both research areas and researchers.*

**RE:**  Since we agree to that proposal, we have added a final paragraph about further research on the issue of OER use.
